# Myocardial infarction differentially alters sphingolipid levels in plasma, erythrocytes and platelets of the rat

**DOI:** 10.1007/s00395-012-0294-0

**Published:** 2012-09-09

**Authors:** Małgorzata Knapp, Małgorzata Żendzian-Piotrowska, Agnieszka Błachnio-Zabielska, Piotr Zabielski, Krzysztof Kurek, Jan Górski

**Affiliations:** 1 Department of Cardiology, Medical University of Białystok, Skłodowskiej-Curie 24a, 15-276 Białystok, Poland; 2 Department of Physiology, Medical University of Białystok, Mickiewicza 2C, 15-089, Białystok, Poland

**Keywords:** Myocardial infarction, Sphingolipids, Plasma, Erythrocytes, Platelets, Rat

## Abstract

Three bioactive sphingolipids, namely sphingosine-1-phosphate (S1P), ceramide (CER) and sphingosine (SPH) were shown to be involved in ischemia/reperfusion injury of the heart. S1P is a powerful cardioprotectant, CER activates apoptosis and SPH in a low dose is cardioprotective whereas in a high dose is cardiotoxic. The aim of the present study was to examine effects of experimental myocardial infarction on the level of selected sphingolipids in plasma, erythrocytes and platelets in the rat. Myocardial infarction was produced in male Wistar rats by ligation of the left coronary artery. Blood was taken from the abdominal aorta at 1, 6 and 24 h after the ligation. Plasma, erythrocytes and platelets were isolated and S1P, dihydrosphingosine-1-phosphate (DHS1P), SPH, dihydrosphingosine (DHS) and CER were quantified by means of an Agilent 6460 triple quadrupole mass spectrometer using positive ion electrospray ionization source with multiple reaction monitoring. The infarction reduced the plasma level of S1P, DHS1P, SPH and DHS but increased the level of total CER. In erythrocytes, there was a sharp elevation in the level of SPH and DHS early after the infarction and a reduction after 24 h whereas the level of S1P, DHS1P and total CER gradually increased. In platelets, the level of each of the examined compounds profoundly decreased 1 and 6 h after the infarction and partially normalized in 24 h. The results obtained clearly show that experimental heart infarction in rats produces deep changes in metabolism of sphingolipids in the plasma, platelets and erythrocytes.

## Introduction

It is well documented that certain bioactive sphingolipids, especially sphingosine-1-phosphate (S1P) and ceramide (CER) exert powerful effects on the heart response to experimental ischemia/reperfusion (I/R) injury. Interestingly, each of them exerts different effects. Extracellular S1P is a very powerful cardioprotectant. It was shown to increase viability of isolated cardiomyocytes subjected to hypoxia or ischemia/reperfusion [27, 48, 60]. Also, it was reported to reduce the infarct size and left ventricular end diastolic pressure (LVEDP) and to accelerate recovery of the left ventricular developed pressure (LVDP) in isolated, perfused rat and mouse hearts exposed to ischemia/reperfusion [26, 34, 49, 55]. S1P is claimed to be the principal mediator of ischemic pre- and post-conditioning [24, 25, 54]. It binds to the S1P receptors in the plasma membrane and the complex activates G protein [37, 45]. This, in turn, activates Akt kinase [16, 36, 38] and Stat3 [10, 15, 28] which have prosurvival properties. The kinases also play a key role in cardioprotective action of other mediators including TNFα [20, 30]. Very recent contribution indicates on involvement of mitochondrial S1P in cardioprotection [18]. The main source of S1P in the plasma are erythrocytes [9, 19, 40, 45]. Platelets and endothelial cells also contribute to this pool of S1P [29, 51, 57]. I/R procedure was shown to increase the level of CER in the myocardium of rats and rabbits in vivo and in isolated, perfused rat heart [3, 5, 12, 13, 59] and isolated cardiomyocytes [6]. CER is claimed to strongly activate apoptosis in the I/R heart [3, 6]. The data on effect of I/R on behavior of sphingosine (SPH) in isolated, perfused heart are controversial: both elevation [11] and reduction [12] in its level was reported. SPH present in the perfusion medium in a high dose is cardiotoxic during I/R. On the other hand, at physiological concentration, it is cardioprotective [56]. SPH was shown to be involved in myocardial dysfunction resulted from microembolization of the coronary vessels in dogs [50]. It is also suggested that SPH contributes to a protective action of TNFα on cardiac mitochondria [33].

The data presented above indicate that the plasma S1P and SPH concentration may be an important factor affecting the heart function during ischemia and I/R. To-date, there are only two published studies available concerning the plasma concentration of selected sphingolipids after myocardial infarction and they concern humans. In one study, a 50 % reduction in the total plasma concentration of S1P ąnd dihydrosphingosine 1-phosphate (DHS1P) early after the infarction and in 5 days later was reported. The concentration of SPH, CER and dihydrosphingosine (DHS) remained stable [31]. In the other study [44], the total plasma S1P concentration after the infarction depended on the time after the event: it was stable at <2 h, elevated in 2-12 h and returned to the control, thereafter. Interestingly, the HDL-bound concentration of plasma S1P in patients early after myocardial infarction was found to be reduced whereas the concentration of non-HDL-bound S1P was elevated.

At present, there are no data on the effect of myocardial infarction on metabolism of sphingolipid intermediates in different blood compartments. The aim of the present study was to examine the level of S1P, SPH, DHS, DHS1P and CER in plasma, erythrocytes and platelets after ligation of the left coronary artery in rats.

## Methods

The experimental procedure was approved by the Ethical Committee on the Animal Research at the Medical University of Białystok. Male Wistar rats, 200–220 g body weight (42–48 day old) were used. There were no age differences between the groups.

### The procedure of ligation of the left coronary artery

The surgery was performed under thiopental anesthesia (80 mg/100 g of body weight). The thoracic cavity was opened in the left fifth intercostal space. The lungs were ventilated by frequent air puffs administered manually by means of a small rubber balloon connected with the rat’s nose by means of a plastic tube. The heart was exteriorized, the pericardium was cut open and the left coronary artery was ligated with a 6-0 monofilament polypropylene suture thread (Surgipro). The heart was placed back in the thoracic cavity, the wound was sutured and the rat started to breath spontaneously. After the surgery the rats were placed in their home cages. The same procedure was applied during the sham surgery with the exception that the coronary artery was not ligated. The rate of survival of the rats subjected to the myocardial infarction was 60 %. In separate rats (*N* = 3), the ischemic area was determined 24 h after ligation of the left coronary artery using Evans Blue [35]. The area encompassed 35, 36 and 39 % of the left ventricle.

### Ligation of the femoral artery

The rats were anaesthetized as above. A small (~5 mm) incision of the skin in the inguinal fossa was made, the femoral artery was separated bluntly from the vein and the artery was tied up. The incision was secured with two sutures. Since the incision was so small sham surgery was not performed. 6 h after ligation of the artery the rats were anaesthetized again and the blood was taken and processed as described below. The foot of the leg was pale and cold at the time of blood collection.

### Blood collection and fractionation

Blood samples were drawn through a catheter inserted into an abdominal aorta. Sodium citrate was used as an anticoagulant. Immediately after sampling, 4 ml of blood was centrifuged at 300×*g* for 10 min at room temperature and platelet-rich plasma was transferred to a fresh plastic tube. The leukocyte-rich buffy coat was thoroughly removed. Separated erythrocytes were suspended in 0.9 % NaCl, centrifuged at 800×*g* for 10 min and the upper layer and the remaining buffy coat were discarded. Red blood cells were then resuspended in 2 ml of 0.9 % NaCl and flash frozen in liquid nitrogen. Platelet-rich plasma was centrifuged at 1,000×*g* for 10 min to isolate platelets. Supernatant was then transferred to a fresh plastic tube and centrifuged at 5,000×*g* for 10 min to obtain platelet-free plasma. Isolated platelets were washed with platelet wash buffer (5 mM KH_2_PO_4_, 5 mM Na_2_HPO_4_, 0.1 M NaCl, 1 % glucose, 0.63 % sodium citrate, pH 6.6), suspended in 0.3 ml of PBS, and flash frozen in liquid nitrogen. All samples were stored at −80 °C until analysis.

The erythrocyte and platelet variables were measured in the blood using ABX Micros ABC Vet analyzer (Horiba).

The plasma level of cardiac rat troponin I isoform (TNNI3) was measured with the use of commercially available ELISA assay (ELISA Kit for rat Troponin I Type 3) according to manufacturer’s guidelines (USCN Life Science & Technology Company, TX, USA).

The protein content in platelet samples and hemoglobin content in erythrocyte suspension was measured with the use of BCA-1 kit and Drabkin’s reagent, respectively (both from Sigma-Aldrich, St. Louis, MO, USA).

### Determination of sphingolipids

Standards utilizing 18C-sphingoid bases: sphingosine d18:1 (SPH) and dihydrosphingosine d18:0 (DHS), sphingosine-1-phosphate d18:1 (S1P), dihydrosphingosine-1-phosphate (DHS1P), d18:1/14:0-Cer—ceramides containing myristic acid (C14:0-Cer), d18:1/16:0-Cer—ceramides containing palmitic acid (C16:0-Cer), d18:1/17:0-Cer—ceramides containing margaric acid (C17:0-Cer)—internal standard for ceramides, d18:1/18:0-Cer—ceramides containing stearic acid (C18:0-Cer), d18:1/18:1-Cer— ceramides containing oleic acid (C18:1-Cer), d18:1/20:0-Cer—ceramides containing arachidic acid (C20:0-Cer), d18:1/24:0-Cer—ceramides containing lignoceric acid (C24:0-Cer), d18:1/24:1-Cer—ceramides containing nervonic acid (C24:1-Cer) as well as internal standards utilizing 17C-sphingoid bases: sphingosine (d17:1-SPH)—internal standard for sphingosine and dihydrosphingosine, sphingosine-1-phosphate (d17:1-S1P)—internal standard for sphingosine-1-phosphate and dihydrosphingosine 1-phosphate, were purchased from Avanti Polar Lipids. Dihydrosphingosine-1-phosphate (d18:0) was purchased from Sigma-Aldrich (St. Louis, MO). HPLC grade methanol, water, formic acid, ammonium formate and ethanol were purchased from Sigma-Aldrich (St. Louis, MO). Sphingolipids were extracted from the plasma, erythrocytes and platelets by the use of the extraction mixture composed of isopropanol:water:ethyl acetate (35:5:60; v:v:v). The level of sphingolipids was determined according to [8]. The following sphingolipids were quantified: sphingosine, sphingosine-1-phosphate, dihydrosphingosine (sphinganine), dihydrosphingosine-1-phosphate, ceramide C14:0, C16:0, C18:1, C18:0, C20:0, C22:0 C24:1 and C24:0. Sphingolipids were analyzed by means of an Agilent 6460 triple quadrupole mass spectrometer using positive ion electrospray ionization (ESI) source with multiple reaction monitoring (MRM). Chromatographic separation was performed using an Agilent 1290 Infinity Ultra Performance Liquid Chromatography (UPLC). The analytical column was a reverse-phase Zorbax SB-C8 column 2.1 × 150 mm, 1.8 μm. Chromatographic separation was conducted in binary gradient using 2 mM ammonium formate, 0.15 % formic acid in methanol as Solvent A and 1.5 mM ammonium formate, 0.1 % formic acid in water as Solvent B at the flow rate of 0.4 ml/min.

### Determination of sphingosine kinase activity

Sphingosine kinase (SPHK) activity in blood platelets and erythrocytes was measured according to Vessey et al. [53] with minor modifications. Briefly, isolated platelets or erythrocytes were sonicated in 100 mM Tris HCl, pH = 8.0 for 30 s on ice. An aliquot of the sample corresponding to approx 100 μg of platelet protein or erythrocyte hemoglobin was incubated for 15 min. in assay buffer (100 mM Tris HCl, pH = 8.0, 250 mM KCl, 0.05 % Triton X-100, 20 mM MgCl_2_) with 10 mM ATP and 5 μM of [3-^3^H]-sphingosine as substrates (approx. 400 DPM/pmol American Radiolabeled Chemicals Inc. St. Louis, MO, USA). Sodium fluoride, semicarbazide hydrochloride and β-glycerophosphate were added to a final concentration of 10 mM, 2 mM and 40 mM, respectively, to inhibit activity of lipid phosphatases and sphingosine-1-phosphate lyase. The reaction was terminated by addition of ice-cold methanol followed by chloroform and 200 mM EDTA. After vortexing/centrifugation aqueous phase containing water-soluble ^3^H-sphingosine-1-phosphate was collected. Radioactivity was measured by Packard 1900TR liquid scintillation counter. Activity of the enzyme was expressed in pmol of S1P/min/mg of platelet protein or erythrocyte hemoglobin.

### Statistical analysis

All data are presented as mean ± SD. Data were analyzed by ANOVA with Tukey HSD (Honestly Significant Difference) post hoc test for unequal sample size. *p* < 0.05 is regarded as significant. Eight rats were used for each time point in either group with the exception of the sham operated group after 1 h (*N* = 7).

## Results

### The erythrocyte and platelet variables (Table 1)

Neither ligation of the left coronary artery nor sham surgery affected the erythrocyte and platelets variables.

**Table 1:** Erythrocyte and platelet variables in 6 h after ligation of the left coronary artery in the rat

Variable (unit)	Control	Sham operated	Ligation of the coronary artery
RBC (10^6^/μL)	6.69 ± 0.60	6.66 ± 0.50	6.72 ± 0.64
HGB (g/dL)	12.37 ± 0.51	12.96 ± 0.26	12.68 ± 0.59
HCT (%)	34.70 ± 2.38	36.80 ± 1.78	35.84 ± 5.70
MCV(fL)	54.30 ± 1.21	54.12 ± 5.05	52.88 ± 2.62
MCH (pg)	19.39 ± 0.43	19.75 ± 0.91	19.50 ± 0.41
MCHC (g/dL)	35.74 ± 1.40	36.80 ± 1.51	36.80 ± 1.50
PLT (10^3^/μL)	920.29 ± 106.81	972.39 ± 31.58	962.71 ± 36.08
MPV (fL)	6.21 ± 0.82	6.49 ± 0.44	6.25 ± 0.45

### Troponin T (Fig. 1)

Troponin T was undetectable in the control and sham operated rats. It increased gradually after ligation of the left coronary artery so that after 6 h it was significantly higher (*p* < 0.001) than after 1 h and after 24 h it was higher (*p* < 0.05) than after 6 h.

**Fig. 1:**
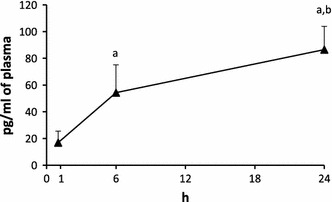
The impact of myocardial infarction on the plasma concentration of rat cardiac troponin I (TNNI3, myocardial damage marker). Values from control and sham-operated groups were below the level of detection, and are not shown. *Black vertical bars* represent SD (*n* = 6 per group, with the exception of 24 h post-infract, *n* = 5). *a* *p* < 0.001 as compared with 1 h post-infarct group value, *b* *p* < 0.05 as compared with 6 h post-infarct group value

### Sphingolipids

The control levels of sphingolipids are presented in Table 2.

**Table 2:** The control values of particular sphingolipids in the blood compartments

Compound	Plasma^a^	Erythrocytes^b^	Platelets^c^
SPH	14.10 ± 1.08	3.65 ± 0.36	0.64 ± 0.06
DHS	9.08 ± 0.71	2.22 ± 0.31	0.35 ± 0.03
S1P	198.76 ± 10.87	5.45 ± 0.41	5.65 ± 0.33
DHS1P	49.29 ± 1.97	5.29 ± 0.45	2.64 ± 0.19
CER	2.03 ± 0.1^d^	21.03 ± 1.43	216.63 ± 22.4

*SPH* sphingosine, *DHS* dihydrosphingosine, *S1P* sphingosine-1-phosphate, *DHS1P* dihydrosphingosine-1-phosphate, *CER* ceramide

The numbers are mean ± SD, *N* = 8

^a^pmol/ml

^b^pmol/mg of Hb

^c^pmol/mg of protein

^d^nmol/ml

### Effect of ligation of the left coronary artery

#### Sphingosine-1-phosphate (S1P) (Fig. 2)

The level of S1P in the plasma after ligation of the artery (Infarct) decreased by 19.4 % in 1 h, by 57.7 % in 6 h and returned to the control value in 24 h. In sham operated rats (Sham) the level of S1P decreased in 6 h but it was higher from the respective value in Infarct. It did not return to the control value in 24 h.

**Fig. 2:**
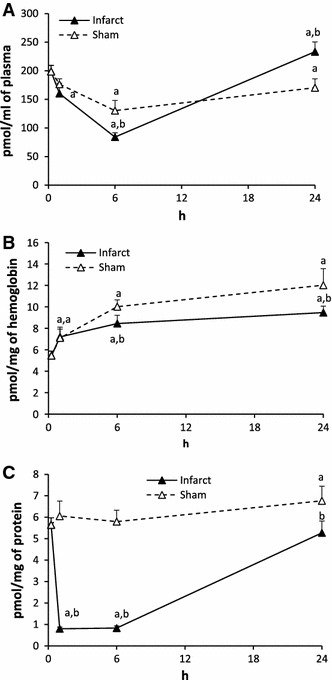
The impact of myocardial infarction (*black triangles*) or sham operation (*open triangles*) on the level of sphingosine-1-phosphate (S1P) in blood plasma (**A**), erythrocytes (**B**) and platelets (**C**). *Black vertical bars* represent SD. When bars not visible, SD is smaller than the size of the marker. *a* *p* < 0.05 as compared to the control, *b* *p* < 0.05 as compared to appropriate sham group

The S1P level in erythrocytes was significantly higher at each time point both in Infarct and Sham as compared to the control value.

In platelets, the level of S1P in Infarct dropped down dramatically in 1 and 6 h (by 85.5 and 85.2 %, respectively) and returned to the control value in 24 h. In Sham, the level of S1P in 1 and 6 h did not differ whereas in 24 h was higher from the control value.

#### Sphingosine (SPH, Fig. 3)

The plasma level of SPH in Infarct in 1 and 6 h was 2.8 and 3.5 times lower from the control, respectively. It returned to normal in 24 h. In Sham, the level of SPH in 1 and 6 h was lower than the control but higher from the respective value in Infarct.

**Fig. 3:**
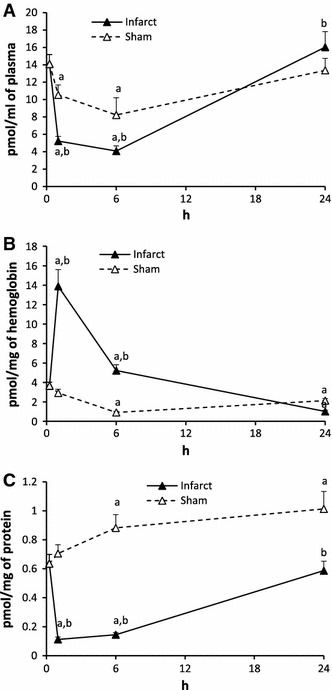
The impact of myocardial infarction (*black triangles*) or sham operation (*open triangles*) on the level of sphingosine (SPH) in blood plasma (**A**), erythrocytes (**B**) and platelets (**C**). *Black vertical bars* represent SD. When bars not visible, SD is smaller than the size of the marker. *a* *p* < 0.05 as compared to the control, *b* *p* < 0.05 as compared to appropriate sham group

The level of SPH in erythrocytes of Infarct in 1 h increased 4 times over the control. In 6 h it was 1.5 times higher whereas in 24 h it dropped down and was 3.5 times lower than the control. In Sham, the content of SPH in 6 h and 24 h was lower than in the control.

The level of SPH in the platelets of the Infarct in 1 h was nearly 6 times and in 6 h over 4 times lower than the control and in 24 h it returned to the control. In Sham, the level of SPH in 6 and 24 h was significantly higher than the control.

#### Dihydrosphingosine (DHS, Fig. 4)

The level of DHS in the plasma of Infarct in 1 h was over 5 times lower and in 6 h was over 7 times lower than the control. It returned to the control in 24 h. Sham surgery did not affect the level of DHS.

**Fig. 4:**
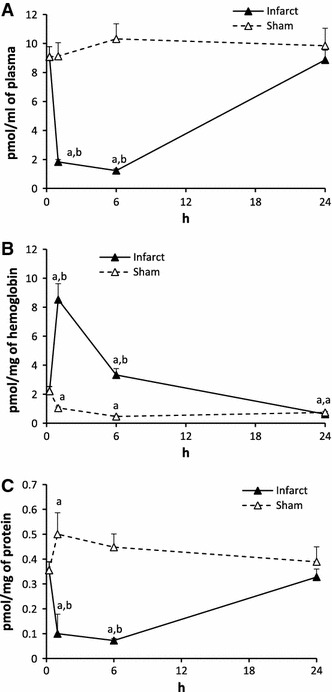
The impact of myocardial infarction (*black triangles*) or sham operation (*open triangles*) on the level of dihydrosphingosine (DHS) in blood plasma (**A**), erythrocytes (**B**) and platelets (**C**). *Black vertical bars* represent SD. When bars not visible, SD is smaller than the size of the marker. *a* *p* < 0.05 as compared to the control, *b* *p* < 0.05 as compared to appropriate sham group

In erythrocytes, the level of DHS in Infarct in 1 h increased 3.8 times over the control. Then, in 6 h it decreased to a value 1,5 times higher than the control whereas in 24 h it was 3.6 times lower than the control. In Sham, the level of DHS was markedly lower than the control at each time point.

In platelets, the level of DHS in Infarct in 1 and 6 h was over 3 times lower than the control and returned to the normal after 24 h. In Sham, the level of DHS increased significantly in 1 h and then returned to normal.

#### Dihydrosphingosine-1-phosphate (DHS1P, Fig. 5)

The level of DHS1P in plasma of Infarct decreased considerably in 1 and 6 h and returned to normal in 24 h. In Sham, the concentration of the compound in the experimental groups did not differ from the control value.

**Fig. 5:**
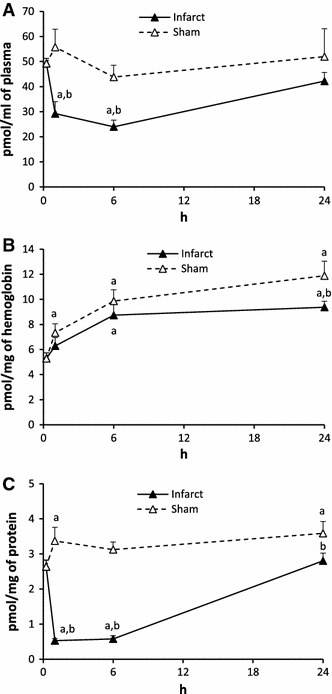
The impact of myocardial infarction (*black triangles*) or sham operation (*open triangles*) on the level of dihydrosphingosine-1-phosphate (DHS1P) in blood plasma (**A**), erythrocytes (**B**) and platelets (**C**). *Black vertical bars* represent SD. When bars not visible, SD is smaller than the size of the marker. *a* *p* < 0.05 as compared to the control; *b* *p* < 0.05 as compared to appropriate sham group

The level of DHS1P in erythrocytes of Infarct in 6 and 24 h was significantly higher that the control. In Sham, the level of DHS1P was significantly higher than the control at each time point.

In platelets, the level of DHS1P in Infarct in 1 and 6 h was about 5 times lower than the control. In sham, it was elevated in 1 and 24 h compared to the control.

#### Total ceramide (CER, Fig. 6)

The plasma level of total CER in Infarct was elevated in 1 and 6 h whereas in Sham it remained stable at each time point.

**Fig. 6:**
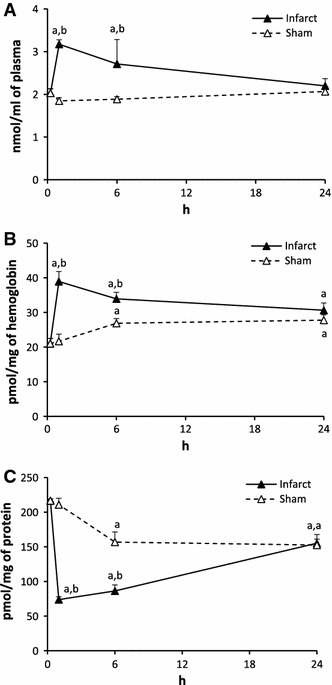
The impact of myocardial infarction (*black triangles*) or sham operation (*open triangles*) on the total level of ceramide (CER) in blood plasma (**A**), erythrocytes (**B**) and platelets (**C**). *Black vertical bars* represent SD. When bars not visible, SD is smaller than the size of the marker. *a* *p* < 0.05 as compared to the control, *b* *p* < 0.05 as compared to appropriate sham group

The level of total CER in erythrocytes in Infarct was markedly higher than the control at each time point whereas in Sham, the level was elevated in 6 and 24 h only.

In platelets, the level of total Cer decreased dramatically in Infarct in 1 and 6 h and returned partially to the control in 24 h. In Sham the level of total Cer in 1 and 6 h was also reduced though to much lesser degree than in Infarct.

##### Individual ceramides (Tables 3, 4, 5)

Because of a great number and diversity of data, the levels of individual ceramides are shown in the Tables 3, 4, 5 and are not presented in detail here.

**Table 3:** Effect of myocardial infarction on the level of individual ceramides in plasma of the rat

Ceramide	Control	1 h		6 h		24 h	
C14	220.71 ± 17.75	I	219.75 ± 26.79	I	4.10 ± 0.65^a,b^	I	192.27 ± 17.38
		S	181.98 ± 28.01	S	209.32 ± 40.82	S	232.87 ± 40.95
C16	278.60 ± 20.39	I	555.60 ± 23.96^a,b^	I	350.27 ± 34.95^a,b^	I	387.60 ± 41.83^a,b^
		S	327.12 ± 41.03	S	428.48 ± 44.75^a^	S	303.53 ± 40.92
C18	3.55 ± 0.30	I	3.73 ± 0.34^b^	I	3.83 ± 0.55	I	6.29 ± 1.59^a,b^
		S	5.21 ± 0.60^a^	S	5.12 ± 0.67^a^	S	4.19 ± 0.39
C18:1	4.15 ± 0.30	I	4.36 ± 0.57	I	4.77 ± 0.41	I	5.57 ± 0.62^a^
		S	4.08 ± 0.94	S	4.08 ± 0.39	S	4.56 ± 0.83
C20	4.80 ± 0.45	I	7.10 ± 0.42^a,b^	I	5.79 ± 0.76	I	6.21 ± 0.52^a^
		S	4.60 ± 0.40	S	5.81 ± 1.08	S	5.34 ± 0.69
C22	92.68 ± 8.21	I	135.12 ± 14.87^a,b^	I	166.06 ± 19.63^a,b^	I	122.69 ± 11.93^a,b^
		S	80.44 ± 7.93	S	65.52 ± 15.51^a^	S	67.81 ± 12.41
C24	1207.13 ± 83.08	I	1928.42 ± 94.92^a,b^	I	1894.13 ± 523.12^a,b^	I	1163.07 ± 105.89
		S	1089.15 ± 108.61	S	1016.76 ± 84.22	S	1249.32 ± 80.48
C24:1	217.76 ± 8.36	I	318.56 ± 34.98^a,b^	I	282.53 ± 12.16^a,b^	I	312.85 ± 38.31^a,b^
		S	153.77 ± 19.40^a^	S	149.51 ± 15.07^a^	S	196.48 ± 20.92

The total level of ceramide is presented at the Table 2 and the Fig. 5

pmol/ml, values are mean ± SD

*I* infarct, *S* sham operated, *N* = 8 with the exception of sham 1 h where *N* = 7

^a^ *p* < 0.05 as compared to the control

^b^ *p* < 0.05 as compared to the respective value in the Sham group

**Table 4:** Effect of myocardial infarction on the level of individual ceramides in erythrocytes (pmol/mg Hb) of the rat

Ceramide	Control	1 h		6 h		24 h	
C14	0.068 ± 0.006	I	0.123 ± 0.011^a,b^	I	0.080 ± 0.010^b^	I	0.078 ± 0.006
		S	0.058 ± 0.007	S	0.056 ± 0.005	S	0.075 ± 0.009
C16	5.226 ± 0.406	I	8.979 ± 0.877^a,b^	I	6.108 ± 0.610	I	5.838 ± 0.644
		S	5.139 ± 0.606	S	5.792 ± 0.499	S	6.112 ± 0.499
C18	0.194 ± 0.016	I	0.594 ± 0.063^a,b^	I	0.440 ± 0.028^a,b^	I	0.256 ± 0.027^a^
		S	0.198 ± 0.032	S	0.264 ± 0.022a	S	0.239 ± 0.021
C18:1	0.082 ± 0.007	I	0.160 ± 0.016^a,b^	I	0.116 ± 0.008^a,b^	I	0.089 ± 0.007
		S	0.087 ± 0.007	S	0.086 ± 0.007	S	0.086 ± 0.009
C20	0.103 ± 0.009	I	0.227 ± 0.024^a,b^	I	0.207 ± 0.025^a,b^	I	0.154 ± 0.015^a,b^
			0.129 ± 0.016	S	0.137 ± 0.014^a^	S	0.127 ± 0.012
C22	1.894 ± 0.073	I	3.561 ± 0.454^a,b^	I	2.850 ± 0.188^b^	I	2.468 ± 0.242^a^
		S	2.055 ± 0.267	S	2.285 ± 0.147	S	2.296 ± 0.264
C24	10.627 ± 1.158	I	18.982 ± 1.831^a^	I	19.232 ± 1.459^a^	I	17.309 ± 1.154^a^
		S	11.022 ± 1.339	S	14.814 ± 0.886^a^	S	15.451 ± 1.415^a^
C24:1	2.835 ± 0.193	I	6.317 ± 0.695^a,b^	I	4.901 ± 0.306^a,b^	I	4.441 ± 0.458^a^
		S	2.996 ± 0.333	S	3.445 ± 0.327	S	3.398 ± 0.256

Total level of total ceramide is presented at the Table 2 and at the Fig. 5

Values are mean ± SD

*I* infarct, *S* sham operated

*N* = 8 with the exception of sham 1 h where *N* = 7

^a^ *p* < 0.05 as compared to the control

^b^ *p* < 0.05 as compared to the respective value in the Sham group

**Table 5:** Effect of myocardial infarction on the level of individual ceramides in platelets (pmolg/mg protein) of the rat

Ceramide	Control	1 h		6 h		24 h	
C14	0.92 ± 0.08	I	0.26 ± 0.11^a,b^	I	0.59 ± 0.45	I	0.78 ± 0.06
		S	0.93 ± 0.10	S	0.78 ± 0.04	S	0.78 ± 0.06
C16	74.66 ± 9.96	I	11.42 ± 0.48^a,b^	I	29.93 ± 4.33^a,b^	I	57.45 ± 6.41^a^
		S	82.17 ± 7.40	S	59.12 ± 7.32^a^	S	54.84 ± 3.31^a^
C18	9.74 ± 0.78	I	3.33 ± 0.25^a,b^	I	2.65 ± 0.37^a,b^	I	6.89 ± 0.81^a^
		S	8.66 ± 1.17	S	6.54 ± 0.58^a^	S	6.36 ± 0.57^a^
C18:1	1.33 ± 0.16	I	0.92 ± 0.15^a,b^	I	0.71 ± 0.05^a,b^	I	0.71 ± 0.04^a,b^
		S	1.32 ± 0.09	S	0.89 ± 0.09^a^	S	0.89 ± 0.11^a^
C20	3.31 ± 0.25	I	1.97 ± 0.17^a^	I	1.52 ± 0.20^a^	I	1.68 ± 0.40^a^
		S	2.38 ± 0.32^a^	S	1.35 ± 0.10^a^	S	1.35 ± 0.42^a^
C22	21.06 ± 2.22	I	10.45 ± 0.58^a,b^	I	9.00 ± 3.28^a^	I	15.43 ± 1.53^a,b^
		S	15.59 ± 2.43^a^	S	11.90 ± 1.22^a^	S	11.41 ± 1.09^a^
C24	81.68 ± 10.32	I	36.61 ± 3.66^a,b^	I	30.30 ± 4.26^a,b^	I	54.30 ± 5.46^a^
		S	75.37 ± 7.54	S	60.30 ± 5.94^a^	S	59.53 ± 4.48^a^
C24:1	23.98 ± 2.59	I	8.79 ± 1.03^a,b^	I	11.42 ± 1.42^a,b^	I	17.74 ± 0.60^a^
		S	24.22 ± 2.87	S	15.89 ± 1.37^a^	S	17.13 ± 1.14^a^

Total level of ceramide is presented at the Table 2 and the Fig. 5

Values are mean ± SD

*I* infarct, *S* sham operated

*N* = 8 with the exception of sham 1 h where *N* = 7

^a^ *p* < 0.05 as compared to the control

^b^ *p* < 0.05 as compared to the respective value in the Sham group

#### Activity of sphingosine kinase (SK, Fig. 7)

In erythrocytes of Infarct, SK activity increased in 1 h and it returned to the control, thereafter. In Sham, SK activity was elevated at each time point studied.

**Fig. 7:**
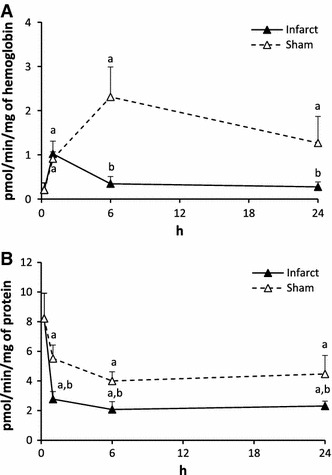
The impact of myocardial infarction (*black triangles*) or sham operation (*open triangles*) on the activity of sphingosine kinase (SK) in erythrocytes (**A**) and platelets (**B**). *Black vertical bars* represent SD (*n* = 6 per group). *a* *p* < 0.05 as compared to the control, *b* *p* < 0.05 as compared to appropriate sham group

In platelets, the activity of SK was reduced in 1 h in both groups and remained further stable. The values in Infarct were lower from the respective values in Sham.

#### Effect of ligation of the femoral artery

**Fig. 8:**
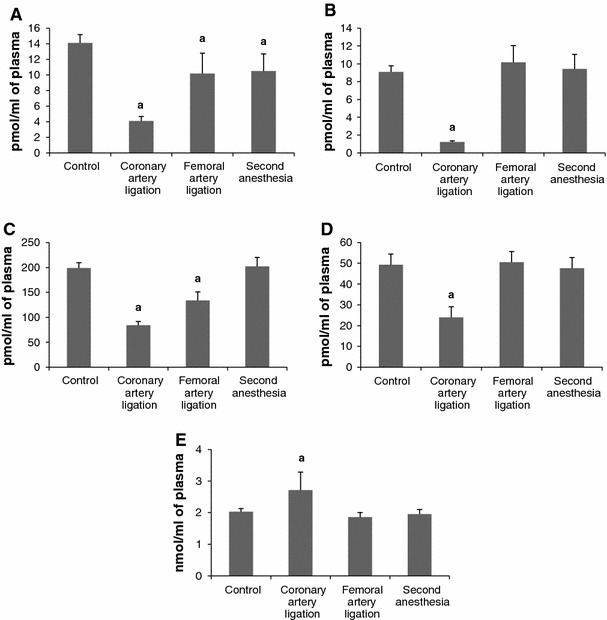
The impact of ligation of the left coronary artery, ligation of the femoral artery and second anesthesia on the plasma level of sphingosine (**A**) dihydrosphingosine (**B**), sphingosine-1-phosphate (**C**), dihydrosphingosine-1-phosphate (**D**) and ceramide (**E**). The blood samples were taken 6 h after ligation of the arteries and after second anesthesia applied in 6 h after the first one. Values represent mean ± SD. *a* *p* < 0.05 as compared to the control

Plasma (Fig. 8). The levels of SPH and S1P were reduced but remained higher from the respective value after ligation of the left coronary artery. The level of DHS, DHS1P and total ceramide remained stable.

**Fig. 9:**
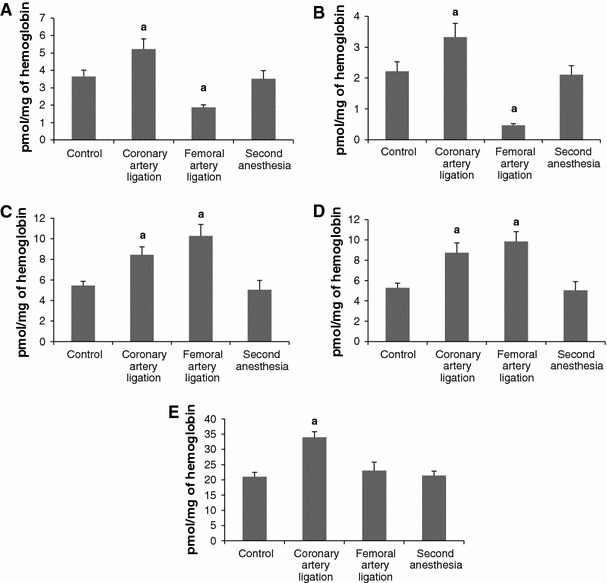
The impact of ligation of the left coronary artery, ligation of the femoral artery and second anesthesia on the level of sphingosine (**A**) dihydrosphingosine (**B**), sphingosine-1-phosphate (**C**), dihydrosphingosine-1-phosphate (**D**) and ceramide (**E**) in erythrocytes. The blood samples were taken 6 h after ligation of the arteries and after second anesthesia applied in 6 h after the first one. Values represent mean ± SD. *a* *p* < 0.05 as compared to the control

Erythrocytes (Fig. 9). The level of SPH and DHS decreased whereas the level of S1P and DHS1P increased compared both to the respective control value and after ligation of the coronary artery. The level of total ceramide remained stable.

**Fig. 10:**
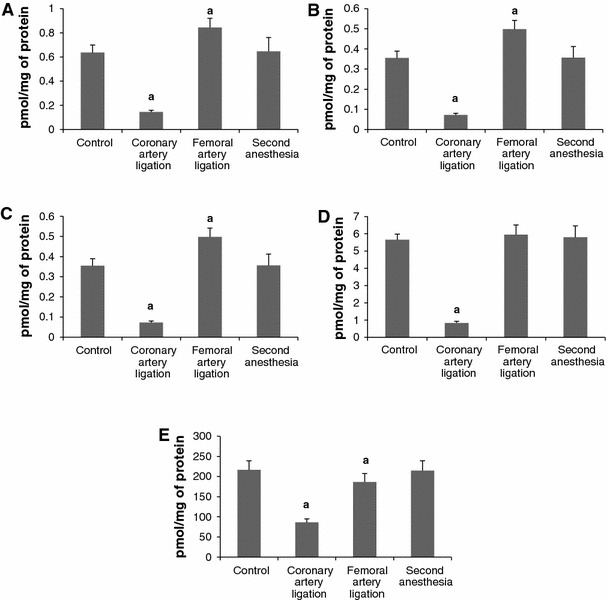
The impact of ligation of the left coronary artery, ligation of the femoral artery and second anesthesia on the level of sphingosine (**A**) dihydrosphingosine (**B**), sphingosine-1-phosphate (**C**), dihydrosphingosine-1-phosphate (**D**) and ceramide (**E**) in platelets. The blood samples were taken 6 h after ligation of the arteries and after second anesthesia applied in 6 h after the first one. Values represent mean ± SD. *a* *p* < 0.05 as compared to the control

Platelets (Fig. 10). The levels of SPH and DHS increased whereas the levels of S1P, DHS1P and total ceramide remained stable.

The levels of individual ceramides in each blood compartment are given in the Table 6.

**Table 6:** Effect of second anaesthesia (A) and ligation of femoral artery (F) on the level of individual ceramides in different blood compartments of the rat

Ceramide	Plasma (pmol/ml)		Erythrocytes (pmol/mg of hemoglobin)		Platelets (pmol/mg of protein)	
C14	C	220.7 ± 17.7	C	0.068 ± 0.006	C	0.924 ± 0.084
	A	235.8 ± 29.6	A	0.074 ± 0.013	A	0.946 ± 0.084
	F	210.6 ± 23.6	F	0.061 ± 0.006	F	0.755 ± 0.117
C16	C	278.6 ± 20.4	C	5.23 ± 0.41	C	74.7 ± 10.0
	A	265.4 ± 29.3	A	5.11 ± 0.72	A	79.0 ± 13.9
	F	298.0 ± 62.1	F	5.51 ± 0.83	F	62.8 ± 14.0
C18	C	3.56 ± 0.30	C	0.194 ± 0.016	C	9.74 ± 0.78
	A	3.68 ± 0.55	A	0.200 ± 0.017	A	9.14 ± 0.51
	F	4.66 ± 0.56^a^.^b^	F	0.232 ± 0.023^a^	F	9.46 ± 1.21
C18:1	C	4.16 ± 0.30	C	0.082 ± 0.007	C	1.34 ± 0.17
	A	4.39 ± 0.70	A	0.090 ± 0.011	A	1.39 ± 0.09
	F	4.29 ± 0.74	F	0.078 ± 0.006	F	1.24 ± 0.18
C20	C	4.81 ± 0.46	C	0.103 ± 0.009	C	3.31 ± 0.25
	A	4.46 ± 0.94	A	0.100 ± 0.015	A	3.31 ± 0.84
	F	5.22 ± 0.47	F	0.125 ± 0.014	F	1.97 ± 0.39^a,b^
C22	C	92.7 ± 8.21	C	1.89 ± 0.07	C	21.0 ± 2.2
	A	90.0 ± 8.53	A	2.38 ± 0.20^a^	A	18.2 ± 1.5
	F	83.2 ± 7.20	F	2.08 ± 0.31	F	16.4 ± 1.6^a^
C24	C	1207.1 ± 83.1	C	10.6 ± 1.16	C	81.6 ± 10.3
	A	1166.6 ± 111.5	A	10.3 ± 1.25	A	79.0 ± 10.9
	F	1054.6 ± 142.7	F	11.5 ± 2.01	F	76.6 ± 9.6
C24:1	C	217.8 ± 8.37	C	2.84 ± 0.19	C	24.0 ± 2.6
	A	185.2 ± 22.5	A	3.14 ± 0.63	A	23.7 ± 3.0
	F	197.7 ± 15.7	F	3.46 ± 0.33	F	17.3 ± 2.5^a,b^

The blood samples were taken 6 h after ligation of the femoral artery and after second anaesthesia applied in 6 h after the first one

The total level of ceramide is presented at the Figs. 8, 9, 10

Values are mean ± SD

*C* control, *A* second anaesthesia, *F* ligation of femoral artery

*N* = 8

^a^ *p* < 0.05 as compared to the control

^b^ *p* < 0.05 as compared to the respective value in the second anaesthesia group

**Fig. 11:**
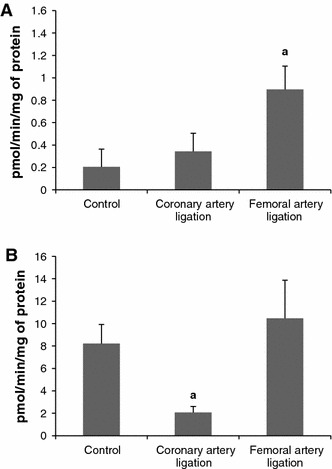
The impact of ligation of the left coronary artery, and ligation of the femoral artery on the activity of sphingosine kinase (SK) in erythrocytes (**A**) and platelets (**B**). The blood samples were taken 6 h after ligation of the arteries.Values represent mean ± SD. *a* *p* < 0.05 as compared to the control

Activity of sphingosine kinase (Fig. 11). Ligation of the artery increased the SK activity in erythrocytes and had no significant effect on the activity in the platelets.

### Effect of anesthesia

Plasma (Fig 8). The anesthesia reduced the level of SPH whereas the other examined variables remained unchanged as compared to the respective control values.

Erythrocytes (Fig. 9) and platelets (Fig. 10). The procedure did not affect the level of either examined sphingolipid.

The levels of individual ceramides in each blood compartment are given in the Table 6.

## Discussion

The results obtained in the present study in rats clearly show that the myocardial infarction produces profound changes in metabolism of examined sphingolipids not only in the plasma but also in erythrocytes and platelets. Moreover, the direction and the size of changes depend both on the species of sphingolipid and the blood compartment. The plasma level of S1P was reduced after the infarction. It is in agreement with previously reported data in humans [30]. It should be noted that, at present, there are no data available on in vivo regulation of behavior of sphingolipids in the plasma. Therefore, it would be premature to hypothesize about a mechanism of the changes in the plasma sphingolipids presented in our study. The enzyme sphingosine kinase (SK) catalyzes phosphorylation of sphingosine to S1P and DHS to DHS1P [9, 23, 47]. The plasma activity of sphingosine kinase is rather low [52]. With the method used in this study [53] the values of the activity of SK in the rat plasma were merely on the level of detection and they are not included in the manuscript. Therefore, changes in the activity in plasma SK after the infarction are not likely to contribute to the reduction in the level of plasma S1P.

The reduction in the plasma level of S1P in Infarct was accompanied by its elevation in erythrocytes and profound reduction in the platelets. Erythrocytes and platelets were shown to possess the enzyme sphingosine kinase and thus are able to synthetize S1P from sphingosine [9, 23, 47]. S1P is catabolized by two enzymes: phosphohydrolase which dephosphorylates the compound and S1P lyase which irreversibly catabolizes it to hexadecenal and phosphoethanolamine. The erythrocytes are the only known cells not to have both enzymes and thus may be considered as the “ideal” store of the compound [23]. In humans, the level of S1P in a single platelet is about 9× higher that in the erythrocyte. However, in a unit of blood the amount of S1P in erythrocytes is much higher than in the platelets which is due to much higher number of erythrocytes as compared to the number of platelets [23]. SK activity in erythrocytes was elevated in 1 h after ligation of the artery and returned to the control value, thereafter. In Sham it was elevated at each time point (Fig. 7). The elevation in SK activity would, at least partially, contribute to elevation of S1P in the cells. Hänel et al. [19] showed that reduction in the plasma/erythrocytes ratio reduces release of S1P. We found no change in the hematocrit, the number of erythrocytes and platelets, and other blood variables after the infarction (Table 1). This excludes a contribution of changes in the cell factor to the level of the examined sphingolipids in the plasma or the erythrocytes during the myocardial ischemia. Erythrocytes were shown to release S1P into plasma. Also, they take up S1P from serum or plasma-free medium [19]. However, since the level of S1P in erythrocytes increased after the infraction, release of the compound into plasma was probably inhibited or at least diminished. It should be added that the mechanism of transport of S1P across the plasma membranes hasn’t yet been recognized. It was hypothesized that ATP cassette transporters are involved in the process [32, 39, 43].

Platelets do not contain S1P lyase but they contain S1P phosphohydrolase [23, 58]. However, it is unlikely that the activity of the latter enzyme increased after the infarction to such an extent that it could account for such a deep reduction in the content of S1P. Human platelets were shown to release S1P upon stimulation [57, 58]. Mouse platelets do not contain S1P but they contain and release DHS1P upon stimulation [14]. It should be stressed that the content of S1P (and DHS1P) in the platelets behave quite opposite to the content in the erythrocytes thus suggesting fundamentally different mechanisms operating in the two type of cells after the infarction. In the platelets, SK activity was reduced in both groups and the reduction in the Infarct was more pronounced than in Sham. Therefore, the reduction in the S1P (and DHS1P) level in platelets in Infarct could be a consequence of reduction of the synthesis of the two compounds. Increased release of the compounds in the infarct should also be taken into account. The coexistence of reduced SK activity and stable level of S1P (and DHS1P) in the platelets in Sham would rather suggest a reduction in release of the compounds.

It is an open question whether the effects of ligation of the coronary artery are specific or whether they can be mimicked by ligation of a peripheral artery. To check this possibility we carried out determinations in the level of sphingolipids in the blood compartments after ligation of the femoral artery. As it can be seen from the Figs. 8, 9, 10 the results obtained differ markedly from the respective data after ligation of the coronary artery. The most striking difference is observed in platelets where the levels of S1P, SPH and DHS increased whereas the level of DHS1P remained stable. The activity of SK in erythrocytes was elevated and this would explain the elevation in S1P level after ligation of the artery. In platelets, the enzyme activity was also elevated but did not reach the level of significance. This degree of elevation in SK activity in the platelets could not probably account solely for elevation in the level of S1P.

The comparison of the results after ligation of the coronary and femoral artery clearly indicates that the exclusion of the each artery affect blood sphingolipid metabolism in a specific way. However, we found no data in the literature to explain the mechanism responsible for the described differences. One may only speculate that ligation of the coronary artery was a much stronger stress than ligation of the femoral artery (see below). Vascular endothelial cells release S1P into plasma. However, neither the rate of secretion nor its extent is recognized, so far [1, 29]. The results obtained after ligation of the femoral artery rather exclude an involvement of endothelium in reduction in the plasma S1P after ligation of the coronary artery. It is interesting that sham surgery also produced numerous changes in the level of the examined compounds. At present, there is no reliable data for explanation of the phenomenon. However, it is well known that a number of stress factors, cytokines and steroid hormones affect sphingolipid metabolism [21, 41]. We examined possible role of second anesthesia (6 h after the first one) and found that it did not affect the level of either sphingolipid (Figs. 8, 9, 10). It may be presumed that thoracotomy and exteriorization of the heart in the sham group was a stress strong enough to produce the observed changes. When we take into account the data in Infarct, in Sham and the data obtained after ligation of the femoral artery we conclude that the sphingolipid signaling system in the blood compartments is very sensitive to different factors. A mechanism creating the changes certainly warrants further investigation. The consequence of the reduction in the plasma S1P concentration after myocardial infarction is not known. The left coronary artery was tightened up permanently so that the blood-borne S1P could not reach the infarcted area. However, the reduction in the plasma S1P concentration may result in reduction of its cardioprotective action in the surrounding area with reduced blood flow as well the intact area.

DHS1P is present in human plasma [2, 19]. It was also reported to be present in mice but not in human erythrocytes and in platelets of both species [14]. We found it in each blood compartment of the rat examined. Its concentration in the mice plasma is much lower than the concentration of S1P [2] and it was presently confirmed in the rat. After the infarction, the level of DHS1P follows changes in the concentration of S1P in each compartment. DHS1P is formed by phosphorylation of dihydrosphingosine (sphinganine) by sphingosine kinase. It also binds to S1P receptors [22]. Its physiological role hasn’t yet been elucidated. It is, therefore, impossible to evaluate functional meaning of the changes in the level of DHS1P after the infarction. However, this indicates that metabolism of the compound in the blood is strongly influenced by the myocardial infarction.

Dihydrosphingosine (DHS) is a precursor of ceramide on its de novo synthesis pathway and sphingosine (SPH) is a product of deacylation of ceramide [17, 41]. DHS was found in minute amounts in mouse plasma and platelets. In humans, it was present only in plasma while sphingosine was present in each compartment [14]. We also previously reported the presence of DHS and SPH in human plasma [31]. Presently, a dramatic reduction in the plasma concentration of both compounds after the infarction was demonstrated. This is in contrast with the data obtained previously in humans where the concentration of the two intermediates remained stable after the infarction [31]. It remains an open question whether this results from interspecies differences or of treatment in humans. SPH (and probably DHS) crosses the plasma membrane [11]. However, the source of the two compounds in plasma is unknown so that it is impossible to speculate on a cause of a reduction in their liberation into plasma after the infarction. The reduction in the plasma concentration of DHS and SPH was accompanied by elevated levels in erythrocytes and profound reduction in their levels in platelets. It is tempting to speculate that increased amounts of DHS and SPH were taken up by erythrocytes early after the infarction what is reflected by elevation in their level in erythrocytes. Thereafter, these precursors were phosphorylated to S1P (and DHS1P, respectively) what resulted in the reduction of their levels in the cells. In platelets, the bases were likely phosphorylated and the phosphorylated derivatives were subsequently released into the plasma. Another possibility is that DHS and SPH were released from the platelets into plasma from where they were taken up by erythrocytes. At present, there is no data on the action of DHS on the heart. As was mentioned in the introduction, SPH, in a low dose, is cardioprotective whereas in a high dose it is cardiotoxic [56]. A reduction in its plasma concentration would likely be beneficial for the undernourished area of the heart.

Ceramide (CER) is a key compound on crossroads of sphingolipid metabolism. In the cell, it is synthetized de novo from serine and palmitoyl CoA in a reaction catalyzed by the enzyme serine palmitoyl transferase and it is formed from sphingomyelin by the action of the enzyme sphingomyelinase [17, 41]. In humans, C24-ceramide is the principal ceramide in plasma and platelets and the amount of C24:1 ceramide is much less abundant. In erythrocytes, the level of both ceramides is almost equal. In mice, C24-1 ceramide constitutes about half of all ceramides in plasma, less than a half in erythrocytes and about 2/3 in platelets [14]. Activation of isolated mouse platelets with thrombin results in severalfold reduction in C24-ceramide and considerable reduction in C24:1-ceramide. It has been suggested that the reduction was due to increased hydrolysis by intracellular ceramidase [14]. It should be added, however, that in another study stimulation of platelets did not affect the activity of the enzyme [42]. In our study, C24-ceramide was also the most abundant ceramide in each examined blood compartment. The infarction elevated the level of total CER in plasma and erythrocytes but reduced it markedly in platelets. In the latter case it resembles the data after stimulation of platelets [14]. Interestingly, the plasma CER concentration was stable after the infarction in humans [31]. Regulation of the level of CER in plasma and the blood cells remains to be recognized. Plasma CER is synthetized in the liver and exported to plasma with lipoproteins. Acid sphingomyelinase and neutral ceramidase are also present in the extracellular space and thus ceramide can be formed and hydrolyzed there. However, the amount of CER formed and removed in the extracellular space is rather small and thus it does not contribute much to the plasma level of this compound [46]. At present, there is also no data which would suggest the mechanism responsible for elevation in the level of CER in erythrocytes after the infarction. As it can be seen in Tables 3, 4, 5, there are numerous changes in behavior in the content of individual ceramides in each examined blood compartment. Neither the direction nor the degree of the changes creates a uniform picture which would allow for interpretation of the individual data. However, it should be mentioned that our knowledge on a mechanism of diversified behavior of particular ceramides or a meaning of it is rather limited. Therefore, this problem will not be further discussed here. We should, at least now, keep in mind that such changes are produced by myocardial infarction.

CER is the intracellular second messenger. It is very active biologically [4, 7, 41]. Naturally occurring long chain ceramides in the plasma cannot cross the plasma membrane. Therefore, one cannot define possible consequence of acute changes in their plasma level.

To summarize, we have found, for the first time, that the experimental heart infarction in the rat produces numerous and profound changes in metabolism of sphingolipids in the plasma, platelets and erythrocytes. It manifests with a reduction in the level of plasma DHS, SPH, S1P and DHS1P and elevation in the level of total CER. In erythrocytes, there was a sharp elevation in the level of SPH and DHS in 1 and 6 h and a reduction in 24 h after the infarction whereas the level of S1P, DHS1P and total ceramide gradually increased. In platelets, the level of the examined compounds profoundly decreased in 1 and 6 h and partially normalized in 24 h after the infarction.
